# Recollection, familiarity, and content-sensitivity in lateral parietal cortex: a high-resolution fMRI study

**DOI:** 10.3389/fnhum.2013.00219

**Published:** 2013-05-23

**Authors:** Jeffrey D. Johnson, Maki Suzuki, Michael D. Rugg

**Affiliations:** ^1^Department of Psychological Sciences, University of MissouriColumbia, MO, USA; ^2^Department of Intelligent Systems, Faculty of Computer Science and Engineering, Khoyama Center for Neuroscience, Kyoto Sangyo UniversityKamigamo-Motoyama, Kita-Ku, Japan; ^3^Center for Vital Longevity, School of Behavioral and Brain Sciences, University of TexasDallas, TX, USA

**Keywords:** episodic memory, recognition memory, content, fMRI, high-resolution MRI, recollection, familiarity

## Abstract

Numerous studies have identified brain regions where activity is consistently correlated with the retrieval (recollection) of qualitative episodic information. This ‘core recollection network’ can be contrasted with regions where activity differs according to the contents of retrieval. The present study used high-resolution fMRI to investigate whether these putatively-distinct retrieval processes engage common versus dissociable regions. Subjects studied words with two encoding tasks and then performed a memory test in which they distinguished between recollection and different levels of recognition confidence. The fMRI data from study and test revealed several overlapping regions where activity differed according to encoding task, suggesting that content was selectively reinstated during retrieval. The majority of recollection-related regions, though, did not exhibit reinstatement effects, providing support for a core recollection network. Importantly, lateral parietal cortex demonstrated a clear dissociation, whereby recollection effects were localized to angular gyrus and confidence effects were restricted to intraparietal sulcus. Moreover, the latter region exhibited a non-monotonic pattern, consistent with a neural signal reflecting item familiarity rather than a generic form of memory strength. Together, the findings show that episodic retrieval relies on both content-sensitive and core recollective processes, and these can be differentiated from familiarity-based recognition memory.

## Introduction

The functional and neural bases of recognition memory have been intensively studied over the past two decades. A significant outcome of this research has been the proposal that recognition judgments are supported by two different memory signals (Mandler, 1980; Yonelinas, 2001, 2002; Wixted and Mickes, 2010). One of these signals supports judgments that are accompanied by the recollection of qualitative information about a prior episode, such as specific details associated with the study item or the context in which it was presented. The other signal supports judgments that are based on an acontextual sense of familiarity. Consistent with this distinction, findings from functional neuroimaging studies have indicated that the neural correlates of recollection and familiarity can be dissociated (for reviews, see Rugg and Yonelinas, 2003; Diana et al., 2007; Skinner and Fernandes, 2007; see Squire et al., 2007, and Wixted et al., 2010, for a dissenting view). Neural correlates of recollection are often reported in the ventral aspect of lateral parietal cortex, in addition to retrosplenial and posterior cingulate cortex, medial prefrontal cortex (PFC), and the hippocampus and parahippocampal cortex (e.g., Henson et al., 1999; Eldridge et al., 2000; Wheeler and Buckner, 2004; Woodruff et al., 2005; Yonelinas et al., 2005; for recent meta-analyses, see Spaniol et al., 2009, and Kim, 2010). The consistency with which recollection-related activity has been reported in these regions has led to the proposal (Johnson and Rugg, 2007; Hayama et al., 2012) that they constitute a ‘core recollection network’ that is engaged regardless of how memory is tested or the nature of the retrieved content. By contrast, neural correlates of familiarity are typically evident in perirhinal cortex, lateral and anterior PFC, the precuneus, and the dorsal aspect of lateral parietal cortex, in the vicinity of the intra-parietal sulcus (e.g., Henson et al., 1999; Yonelinas et al., 2005; Montaldi et al., 2006; also see Kim, 2010).

Successful recollection also engages regions that are putatively outside of the core network, including regions sensitive to the contents of retrieval.^1^ Several studies have reported that recollection is accompanied by ‘cortical reinstatement’—overlap between regions selectively activated during the encoding and the subsequent retrieval of a specific class of study episodes (e.g., Wheeler et al., 2000; Kahn et al., 2004; Johnson and Rugg, 2007; see Rugg et al., 2008, Danker and Anderson, 2010, and Rissman and Wagner, 2012, for reviews). For example, in a study by Johnson and Rugg (2007), subjects studied words that were superimposed either on a landscape scene, in which case the task was to imagine a location in the scene where the object denoted by the word might be found, or on a blank background, when the task was to covertly generate a sentence that incorporated the word. Test items comprised studied and unstudied words under the requirement to endorse items as “remembered” (recollected) if any detail of the encoding episode was retrieved, or “known” (familiar) if recognition was not accompanied by retrieval of episodic details. Relative to items eliciting familiar judgments, activity elicited by recollected items from each study condition overlapped the activity selectively elicited by the respective class of study trials. The findings were interpreted as support for the long-standing proposal (Alvarez and Squire, 1994; McClelland et al., 1995; Rolls, 2000; Shastri, 2002; Norman and O'Reilly, 2003) that retrieval depends upon the recapitulation of the processes and representations that were engaged when the episode was originally experienced.

The aim of the present study was to address a number of outstanding issues regarding the research described above. The first issue concerns the extent to which recollection and familiarity (as operationalized with the “remember/know” procedure) engage brain regions that are anatomically dissociable. Whereas recollection and familiarity have been reported to engage distinct regions (e.g., Henson et al., 1999; Eldridge et al., 2000; Wheeler and Buckner, 2004; Woodruff et al., 2005; Yonelinas et al., 2005), there have been few empirical attempts to directly assess whether and to what extent the neural correlates of the two processes overlap (although, for reviews, see Yonelinas et al., 2005; Vilberg and Rugg, 2007, 2009a). This question is relevant to the debate over whether the recollection-familiarity distinction is better conceived as gradations in an undifferentiated memory signal (hereafter, memory ‘strength’; Donaldson, 1996; Dunn, 2004; Rotello et al., 2005) or as two fully-dissociable signals. Clearly, regions where the neural correlates of the two processes overlap are candidates for signals that support both types of memory. The more extensive the overlap, the stronger is the evidence that recollection and familiarity share neural substrates and, hence, rely on the same processes.

The second issue addressed by the present experiment concerns the functional significance of neural correlates of familiarity. These are almost invariably identified by contrasting the activity elicited by items recognized on the basis of familiarity alone (e.g., items accorded a “know” judgment) with the activity elicited by items for which recognition failed (misses) or that were correctly judged as “new” (correct rejections). Alternatively, in three studies (Vilberg and Rugg, 2007, 2009a,b), neural correlates of familiarity were identified by constraining the analysis to voxels in which recollection effects were absent, thereby unconfounding familiarity and putative ‘strength’ effects (see above). Neither of these analysis strategies sufficiently distinguishes between effects selectively associated with familiarity and effects associated with successful recognition memory more generally—that is, effects that are associated with successful recognition regardless of whether it is based on familiarity or recollection. However, this distinction can be made, at least in principle, given two assumptions: (1) that recollection and familiarity are supported by independent processes, and (2) that the recollection signal is thresholded (Yonelinas, 2001; for an alternative view, see Mickes et al., 2009; Wixted and Mickes, 2010; and Ingram et al., 2012). Using the “remember/know” procedure as an example, under the assumption of independence, items designated with “remember” judgments are largely free to vary in their familiarity levels, since familiarity is not being used as a basis for these responses. The mean familiarity of “remembered” items will consequently be determined by the entire distribution of familiarity levels of the studied items. By contrast, there is a minimum (criterial) level of familiarity that must be attained by an item if it is to be judged as “known”. The mean familiarity of such items should thus be higher than that of “remembered” items (a difference that can be exaggerated by selecting only those non-recollected items that elicit high levels of familiarity; see below). Therefore, in regions where neural activity co-varies with familiarity, the level of activity should be higher for items recognized on the basis of familiarity alone (“known”) than for items that are recollected (“remembered”). A recent magnetoencephalographic (MEG) study utilizing the same logic yielded a non-monotonic effect across familiarity and recollection judgments that was consistent with this prediction (Evans and Wilding, 2012).

The final issue to be addressed relates to the distinction drawn earlier between a core recollection network and regions where retrieval-related activity is content-sensitive. Johnson and Rugg (2007) initially drew this distinction on the basis of the finding that three components of the core network (entorhinal cortex, retrosplenial/posterior cingulate cortex, and left ventral lateral parietal cortex) demonstrated recollection-related activity that was seemingly insensitive to the nature of the recollected content. In that study, though, recollection effects in the hippocampus proper were not identified, leaving open the question of whether retrieval-related hippocampal activity is content-sensitive. Furthermore, the conclusion that the components of the recollection network that were identified are content-insensitive rests on a null finding, at least by the standard of fMRI analyses that are based on detecting mean (smoothed) activity differences (as opposed to pattern-classification analyses; Norman et al., 2006). The goal of the present study was to address these issues with techniques that afforded higher anatomical resolution of BOLD signal changes than previously employed. Thus, the study was performed with an fMRI protocol that used 1.75 mm isotropic voxels in combination with a large-deformation registration method that optimizes across-subject brain alignment (Ashburner, 2007).

We employed an experimental design that combined methods first employed by Yonelinas et al. (2005) with those employed in our prior study (Johnson and Rugg, 2007; also see Johnson et al., 2008a). As in the prior study, subjects studied words in the context of a scene or sentence encoding task. Test items were a mixture of studied and unstudied words, with the requirement to endorse an item as “remembered” if recognition was accompanied by recollection of one of more specific details from the study episode. If recollection failed, items were to be judged with a four-level confidence scale, following Yonelinas et al. (2005); also see Woodruff et al. (2006), Johnson et al. (2009), and Yu and Rugg (2010). This procedure permitted us to operationalize the neural correlates of recollection and familiarity and assess the extent of their overlap, to identify regions where familiarity-related activity exceeded activity associated with successful recollection, and to investigate the overlap between content-sensitive recollection effects and the putative core recollection network.

## Materials and methods

### Subjects

Twenty volunteers were recruited from the student population of the University of California, Irvine (UCI) and paid for their participation. All subjects reported being right-handed and native English speakers, and had normal or corrected-to-normal vision, no history of neurological disease, and no other contraindications for MRI. Informed consent was obtained in accordance with the UCI Institutional Review Board guidelines. The data from four subjects were excluded from all analyses due to insufficient numbers of trials (<10) in at least one condition of interest, leaving 16 subjects (18–24 years old; *M* = 21 years; 10 females).

### Stimuli

The experimental stimuli were drawn from a pool of 260 words denoting single objects (from categories such as tools, furniture, animals, and food; 3–12 letters long, *M* = 6 letters; written frequency, *M* = 18/million; Kucera and Francis, 1967) and a pool of 86 color pictures of outdoor scenes (which excluded buildings, animals, and people). For each subject, three groups of 80 words were randomly selected from the pool. The words from two of the groups were presented in the encoding phase and as old items during the retrieval test phase, while words from the third group served as new test items. For the encoding phase, the words from one group were randomly paired with 80 pictures of scenes. The remaining stimuli were used in a practice phase.

All words were presented in black uppercase 30-point Helvetica font (subtending a visual angle of 0.5° vertically and a maximum angle of 4° horizontally) on a solid yellow rectangle that was slightly larger than the longest word (1° × 4.5°). Word stimuli in the encoding phase appeared near one of the four corners of either a picture of a scene or a solid gray square (each subtending 7° × 7°), so as not to repeatedly obscure any particular part of the scenes. During the retrieval phase, the word stimuli were presented at the center of a single background that was constructed by heavily blurring and pixelating an unused scene and was the same across subjects. A white fixation character (+; 0.5° × 0.5°) was shown during the inter-stimulus intervals and *null* trials (see below) of both phases. All stimuli were displayed centrally on the black background of a screen that was positioned at the head of the magnet bore and viewable through a mirror attached to the head coil.

### Procedure

Subjects received instructions and completed a practice version of the experiment prior to entering the scanner. In the scanner, the experiment consisted of an encoding phase, a retrieval phase, and then the acquisition of anatomical data. The encoding and retrieval phases were each divided into four blocks, corresponding to separate runs of fMRI acquisition with intervening breaks of around 1–2 min.

During the encoding phase, a series of words was presented, with each word superimposed on either a scene or gray background. Subjects were informed that the location of the words (near one of the four corners of the background) was irrelevant to the task. For words superimposed on scenes, subjects were instructed to imagine the object denoted by the word appearing at any location within the scene (the *scene* condition). For words presented on the gray background, subjects were to covertly generate a meaningful sentence incorporating the word (the *sentence* condition). Subjects were instructed to begin the task for each word immediately upon its presentation and to press a button with their right index finger to indicate task completion. The words and backgrounds were displayed until a response was made (up to a maximum of 5 s), after which a fixation character was displayed for 1 s and the next trial began. Intermixed with the word-background trials were 80 null trials, during which the fixation character appeared for 3 s with no response requirement.

The retrieval phase consisted of the presentation of a series of words, each of which either appeared in the encoding phase (*old*) or had not appeared previously (*new*). Subjects were instructed to make one of five judgments to each word (following Yonelinas et al., 2005; Woodruff et al., 2006; Johnson et al., 2009; Yu and Rugg, 2010). When any details associated with a word's presentation during the encoding phase could be *remembered* (*R*), subjects were to press a button with the left index finger. If no details were remembered, subjects used one of four buttons (mapped respectively to the right index through little fingers) to rate their confidence that the word was old or new: *confident old* (*4*), *unconfident old* (*3*), *unconfident new* (*2*), and *confident new* (*1*). The response-hand mappings were reversed for half of the subjects. Response accuracy and speed were given equal emphasis in the instructions. Retrieval trials, consisting of the word and background displayed for 1 s and followed by a fixation character for 2 s, were intermixed with 120 null trials (fixation character for 3 s). Trial order during the encoding and retrieval phases was randomized for each subject, with a limit of four consecutive trials from each condition.

### Imaging parameters

The imaging data were obtained from a Philips Intera Achieva 3 T MR scanner (Philips Medical Systems, Bothell, WA) equipped with an 8-channel SENSE head coil. The fMRI data were acquired using a field-echo EPI pulse sequence sensitive to BOLD contrast (T2^*^-weighted, 70° flip angle, 25 ms TE). Each volume of fMRI data consisted of 41 near-axial images (1.75 mm thick, 0.5 mm gap, anterior-posterior phase-encoding direction) with an in-plane resolution of 1.75 × 1.75 mm (242 × 180 mm FOV, 144 × 144 matrix). The images were oriented parallel to the primary axis of the hippocampus and encompassed the temporal and parietal cortices. A SENSE reduction factor of 2.5 allowed for a relatively short TR of 2.25 s. The self-paced nature of the encoding trials resulted in blocks comprising between 72 and 130 volumes (*M* = 100 across subjects), while each block of the retrieval phase comprised 126 volumes. All blocks were preceded by the acquisition of four TRs of data that were discarded to allow for T1 stabilization. Whole-brain T1-weighted anatomical data were acquired sagittally (240 × 240 mm FOV, 320 × 320 matrix, 0.75 mm isotropic voxels) with a 3D MP-RAGE pulse sequence and a SENSE reduction factor of 1.5.

### Data pre-processing and analysis

The imaging data were processed and analyzed with SPM5 (http://www.fil.ion.ucl.ac.uk/spm) in MATLAB (The MathWorks, Natick, MA). Each volume of the fMRI data was spatially realigned to the first volume of the first encoding block and subsequently to the across-block (encoding and retrieval) mean. The data in each volume were temporally shifted (via sinc interpolation) to the onset of the middle slice and the resulting volumes were co-registered with the anatomical volume. Each subject's anatomical volume was segmented into gray and white matter (Ashburner and Friston, 2005) according to standard tissue probability maps (http://www.loni.ucla.edu/ICBM/). The segmented images were then used with the DARTEL toolbox in SPM5 to create an across-subjects template (Ashburner, 2007). Parameters determined by an affine transformation of the template into Montreal Neurological Institute (MNI) space, along with the DARTEL-based transformation parameters, were then applied to both the anatomical and fMRI data (resampled to 0.75 mm and 1.75 mm isotropic voxels, respectively). The normalized fMRI data were smoothed with a 4 mm FWHM Gaussian kernel.

Prior to analysis of the fMRI data, vectors of onset times of the word stimuli were created for the encoding and retrieval phase conditions. Two vectors, one for the scene condition and the other for the sentence condition, were used for the encoding phase. These vectors took the form of a series of trial-specific boxcar functions extending from word onset to 300 ms prior to button press, which, likely due to the self-paced nature of the encoding phase, we previously found to produce more robust content-related differences (Johnson and Rugg, 2007). For the main analyses reported here, six onset vectors of interest, comprising delta (stick) functions at each word onset, were created to model the retrieval phase data. Two of these vectors corresponded to scene and sentence items that elicited R judgments (R_scene_ and R_sentence_; also see Johnson and Rugg, 2007), while the remaining four vectors represented the different confidence judgments (4, 3, 2, and 1; each of which was collapsed across the scene, sentence, and new conditions). The average numbers of trials contributing to these six conditions ranged from 23 to 51. Two additional retrieval phase vectors coded new items endorsed with R judgments and items eliciting multiple or no button presses, all of which were rare.

The fMRI analysis was based on a two-stage mixed-effects model. In the first stage, the BOLD response associated with each experimental condition was modeled by convolving the vectors described above with a canonical hemodynamic response function (HRF) and its temporal and dispersion derivatives (Friston et al., 1998). The convolved time courses were downsampled at the temporal midpoint of each volume to form the regressors of a general linear model (GLM). Additional covariates corresponding to the six movement parameters determined during realignment (three translations and three rotations) and the within- and across-block means were included in the GLM. The time series for each voxel was high-pass filtered at 1/128 Hz to remove low-frequency noise and scaled to a grand mean of 100 (across voxels and time points). Restricted maximum likelihood (ReML) was used to estimate the regressor-specific parameters and the hyperparameters governing the error covariance. Non-sphericity of the error covariance was accommodated by an AR(1) model, in which the temporal autocorrelation was approximated by pooling over voxels (Friston et al., 2002). In the second stage of analysis, linear contrasts of the estimated parameters were created, treating subjects as a random effect. All of the results reported here are based on parameters corresponding to the canonical HRF, as those based on the derivatives added no meaningful information.

In the main fMRI analyses, a cluster-wise statistical threshold of *P* < 0.05 was used to control for false positives. The minimum cluster extent corresponding to this corrected threshold was determined via Monte Carlo simulation (3dClustSim; http://afni.nimh.nih.gov/pub/dist/doc/program_help/3dClustSim.html) that was based on two factors: the voxel-wise height threshold and the search volume. Unless otherwise noted, the voxel-wise height threshold for individual contrasts was set at *P* < 0.005 (following our previous high-resolution studies; Johnson et al., 2008b; Suzuki et al., 2010, 2011). The search volume consisted of the entire scanned volume of gray matter (213,569 voxels), which was defined on the across-subjects normalized template and smoothed with a 4 mm FWHM Gaussian kernel. The minimum cluster extent based on these parameters was 42 voxels.

The figures display effects either overlaid on the across-subject mean of the normalized T1-weighted images, or projected onto the inflated surface of the standardized PALS-B12 atlas (using Caret 5.61; http://brainvis.wustl.edu/wiki/index.php/Caret:About). Histograms correspond to the across-subject mean parameter estimates (β) from the peak voxel of each effect.

## Results

### Behavioral results

The mean proportions of each retrieval phase judgment are given in Table 1. Old items elicited relatively high proportions of remember (R) judgments, whereas new items frequently elicited unconfident and confident new (2 and 1) judgments. ANOVA of these data, employing factors of item type (scene, sentence, and new) and judgment (R, 4, 3, 2, and 1), revealed a main effect of judgment [*F*_(2.2, 33)_ = 21.80, *P* < 0.001; degrees of freedom corrected according to Greenhouse and Geisser, 1959] and an item type × judgment interaction [*F*_(2.7, 40.4)_ = 48.66, *P* < 0.001]. A follow-up ANOVA of the data for old items only (i.e., scene and sentence) revealed the same effects: *F*_(1.7, 26.2)_ = 45.61, *P* < 0.001, and *F*_(2.9, 43.3)_ = 32.869, *P* < 0.001, respectively, for the item type main effect and interaction with judgment type. Follow-up *t*-tests indicated that R judgments were more frequent for items from the sentence condition than for items from the scene condition [*t*_(15)_ = 8.00, *P* < 0.001], whereas the opposite pattern was evident across 3, 2, and 1 judgments [all *t*_(15)_ > 3.12, all *P* < 0.01]. There was no difference according to item type for 4 judgments.

**Table 1:** **Mean proportions and response times (RTs, in seconds) of judgments for each item type in the retrieval phase**.

**Item type**	**Judgment**				
	**R**	**4**	**3**	**2**	**1**
**PROPORTIONS**					
Scene	0.50 (0.05)	0.14 (0.04)	0.11 (0.03)	0.14 (0.02)	0.11 (0.03)
Sentence	0.66 (0.05)	0.15 (0.04)	0.06 (0.02)	0.06 (0.02)	0.06 (0.02)
New	0.08 (0.03)	0.08 (0.03)	0.13 (0.03)	0.29 (0.04)	0.43 (0.06)
**RTs**					
Scene	1.25 (0.07)	1.51 (0.10)	–	–	–
Sentence	1.22 (0.07)	1.54 (0.09)	–	–	–
New	–	–	–	1.55 (0.12)	1.34 (0.07)
Collapsed	1.24 (0.07)	1.51 (0.08)	1.76 (0.10)	1.61 (0.10)	1.38 (0.07)

R, remember; 4, confident old; 3, unconfident old; 2, unconfident new; 1, confident new. Missing cells indicate item type and judgment combinations where there were too few trials to compute meaningful RT statistics; to accommodate this, RTs in the bottom row are collapsed over all item types. SEM in parentheses.

To facilitate comparisons between the present data and that of previous studies, we computed a measure of memory accuracy (or ‘strength’) as *p*(hit)/(*p*[hit] + *p*[false alarm]) (also see Wixted et al., 2010; Rugg et al., 2012; Yu et al., 2012b). ANOVA of these data, employing item type (scene and sentence) and judgment (R and 4) factors, indicated that both main effects were significant [item type: *F*_(1, 15)_ = 6.25, *P* < 0.05; judgment: *F*_(1, 15)_ = 6.95, *P* < 0.05]. Accuracy was higher for items eliciting R judgments compared to 4 judgments, with this effect being upheld for scene [0.88 vs. 0.70; *t*_(15)_ = 2.51, *p* < 0.05] as well as sentence items [0.91 vs. 0.71; *t*_(15)_ = 2.73, *p* < 0.05]. Consistent with the response proportion data, accuracy of R judgments was also higher for sentence items than for scene items [0.91 vs. 0.88; *t*_(15)_ = 3.32, *p* < 0.01]; however, there was again no item type difference for 4 judgments.

Finally, Table 1 provides the mean response times (RTs) from the retrieval phase. Note that, for the confidence judgments, meaningful RT statistics could not be computed for every item type (also see Table 1 note). For these data, an ANOVA was used first to investigate judgment-related RT differences (R, 4, 3, 2, and 1), regardless of item type (for this analysis, R judgments were also collapsed across scene, sentence, and new). The ANOVA revealed a main effect [*F*_(2.2, 32.3)_ = 28.70, *P* < 0.001], with follow-up *t*-tests indicating significant differences between each pair of judgments [all *t*_(15)_ > 2.32, *P* < 0.05]. Notably, RTs were shortest for R judgments and longest for unconfident old (3) judgments. In a second analysis, we examined RTs for scene and sentence items designated with R and 4 judgments. An ANOVA (with factors of item type and judgment) revealed only a significant main effect of judgment [*F*_(1, 15)_ = 32.77, *P* < 0.001], with RTs being shorter for R judgments. RTs for R and 4 judgments were statistically equivalent across the scene and sentence conditions, as were the corresponding RTs from the encoding phase (scene: *M* = 2.85 s, *SEM* = 0.22; sentence: *M* = 2.73 s, *SEM* = 0.19).

### fMRI results

The fMRI analyses first identified the neural correlates of recollection and familiarity. We then further addressed the functional significance of familiarity correlates, particularly in lateral parietal cortex, by assessing whether the activity exhibited a non-monotonic pattern across recollection and familiarity judgments. Finally, to investigate the extent to which the neural correlates of recollection comprised a ‘core network’, we searched for overlap between that network and regions that exhibited content-sensitivity (scene vs. sentence) at both encoding and retrieval (cf. Johnson and Rugg, 2007).

#### Recollection and familiarity effects

**Figure 1:**
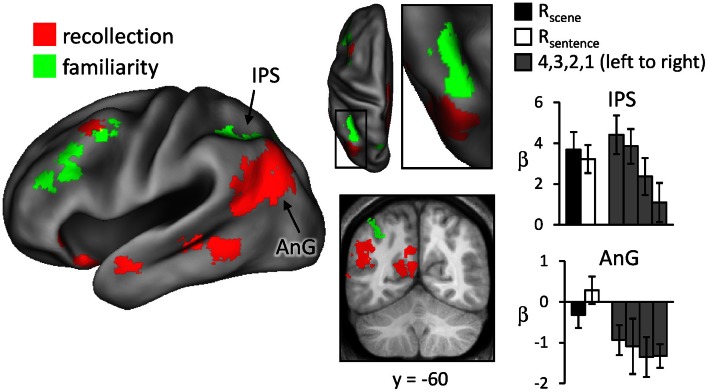
**Neural correlates of recollection (R_scene_/R_sentence_ > 4/3/2/1; red) and familiarity (linear contrast across 4/3/2/1 judgments; green).** Each of these contrasts was exclusively masked with the alternative contrast to demonstrate the anatomical distinction (see text for further details). The effects in left lateral parietal cortex are highlighted in this depiction, but see Figure 2 for analogous effects in other regions. Effects here and in subsequent figures are projected onto a standardized PALS-B12 surface (see Materials and Methods) and overlaid on the across-subjects mean normalized T1-weighted image. Histograms indicate mean parameter estimates (β) from the peak voxel of each effect in angular gyrus (AnG) and intraparietal sulcus (IPS). R (remember); 4, 3, 2, 1 (confidence judgments).

**Figure 2:**
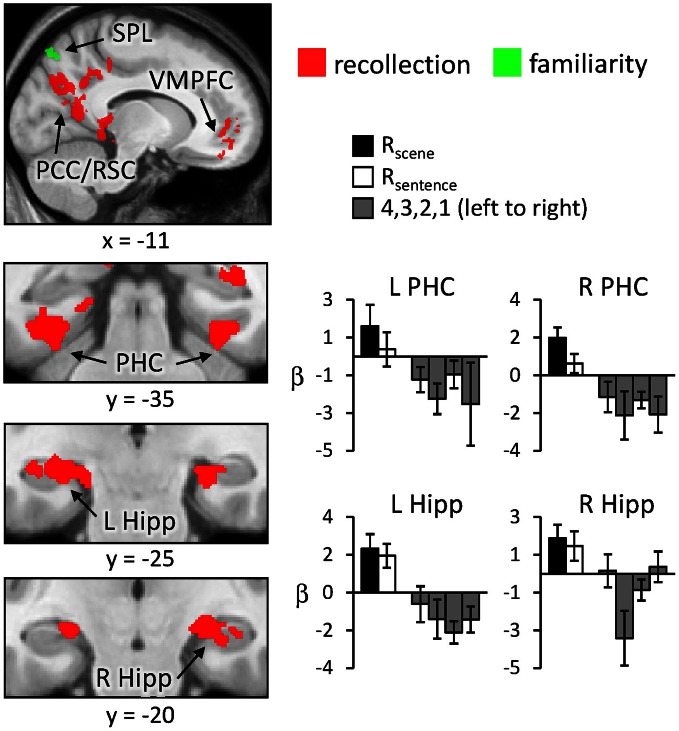
**Neural correlates of recollection (R_scene_/R_sentence_ > 4/3/2/1; red) and familiarity (linear contrast across 4/3/2/1 judgments; green) evident outside of left lateral parietal cortex.** Each of these contrasts was exclusively masked with the alternative contrast to demonstrate the anatomical distinction (see text for further details). Effects in medial temporal lobe (bottom row) are shown on magnified portions of coronal slices, the locations of which are indicated by dotted lines (lower left). SPL (superior parietal lobule), PCC/RSC (posterior cingulate cortex/retrosplenial cortex), VMPFC (ventromedial prefrontal cortex), PHC (parahippocampal cortex), Hipp (hippocampus). R (remember); 4, 3, 2, 1 (confidence judgments).

Regions sensitive to recollection were identified by the contrast of greater activity for R judgments (R_scene_ and R_sentence_) than for the four confidence judgments (4, 3, 2, and 1). To remove any voxels sensitive to familiarity, the outcome of the recollection contrast was exclusively masked with the contrast identifying a parametric increase in activity as a function of recognition confidence (linear contrast weights of 3/1/−1/−3 for the 4/3/2/1 judgments, respectively; at *P* < 0.05).^2^ The results of this analysis are shown in Figures 1 and 2 and detailed in Table 2. As shown in Figure 1, a recollection effect was evident in a region of left ventral parietal cortex that extended across the angular gyrus. As shown in Figure 2, effects were also localized to the medial cortical surface (more prominently on the left). The medial effects were in posterior cingulate and retrosplenial cortex, as well as in a region of medial PFC that extended to the frontal pole and to orbitofrontal cortex. Additionally, recollection effects were evident in several MTL regions bilaterally (see Figure 2), including the body of the hippocampus and posterior parahippocampal cortex (on the left, extending from the aforementioned cluster in retrosplenial cortex).

**Table 2:** **Regions exhibiting effects exclusively related to recollection or familiarity**.

**Region**	**BA**	**Number of voxels**	**Peak coordinates**	**Peak Z score**
**RECOLLECTION EFFECTS**				
L lateral parietal cortex (angular gyrus)	39	1064	−51, −53, 16	5.41
L posterior cingulate/retrosplenial cortex	29/30/31	1454	−5, −53, 11	5.23
R posterior parahippocampal cortex, posterior hippocampus	36	297	33, −51, 5	5.16
R caudate head		86	19, −12, 26	4.96
L medial prefrontal cortex	10/32	860	−7, 61, 4	4.46
L hippocampus (body)		159	−25, −26, −12	4.39
R white matter, ventricle		70	19, −40, 18	4.37
R hippocampus (body)		179	18, −16, −14	4.27
L middle temporal gyrus	21	291	−60, −46, −9	4.18
L middle frontal gyrus	9	58	−37, 11, 46	4.15
L middle temporal gyrus	21	62	−53, −2, −23	4.13
L posterior parahippocampal cortex	36	109	−30, −35, −19	4.05
L posterior cingulate	31	88	−5, −21, 39	3.96
L fornix, thalamus		171	2, −21, 11	3.81
L inferior frontal gyrus	47	72	−35, 33, −12	3.60
L orbitofrontal gyrus	11	42	−32, 21, −19	3.44
**FAMILIARITY EFFECTS**				
L middle frontal gyrus	8/9	132	−46, 11, 46	4.08
L intraparietal sulcus	7/40	198	−37, −61, 46	4.04
L superior parietal lobule	7	48	−12, −77, 54	3.91
L middle frontal gyrus	9/46	248	−42, 30, 33	3.86
R middle frontal gyrus	9/46	47	37, 12, 46	3.76
R anterior cingulated	32	52	2, 35, 30	3.57

Peak coordinates (x, y, z) are in MNI space and rounded to the nearest mm. BA, Brodmann area (approximate); L left; R, right.

Regions where activity was associated with familiarity were identified by the converse of the masking procedure described above. That is, the outcome of the linear contrast across the four confidence levels was exclusively masked with the recollection contrast (R_scene_/R_sentence_ > 4/3/2/1; at *P* < 0.05). As shown in Figure 1 (also see Table 2), activity co-varied positively with familiarity in a region of left lateral parietal cortex that was located primarily on the lateral bank of the intraparietal sulcus. Other familiarity-related effects were evident in precuneus, left and right dorsolateral PFC, and anterior cingulate.

**Figure 3:**
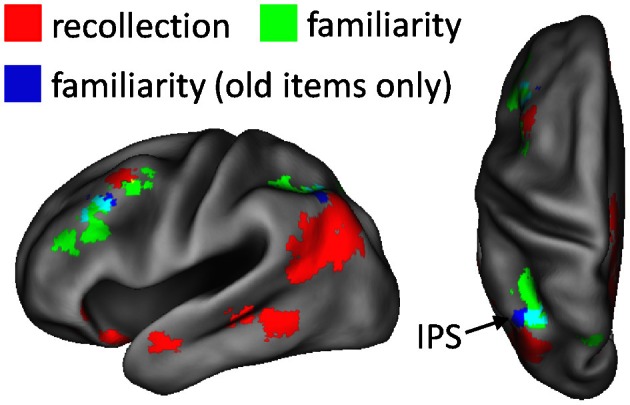
**Regions exhibiting familiarity effects based solely on old test items (blue), shown alongside the neural correlates of recollection (red) and familiarity (green).** Old-item familiarity effects were evident in left intraparietal sulcus (IPS; 56 voxels; peak coordinates = −40, −61, 49; peak Z score = 3.40) and left middle frontal gyrus (60 voxels; peak coordinates = −40, 32, 32; peak Z score = 3.62). The arrow points to overlap (constituting 20 voxels; in cyan) of the familiarity effects in IPS.

As described in section Data pre-processing and analysis, data were collapsed across the content conditions (scene, sentence, and new) for the foregoing analysis, as there were insufficient numbers of items to segregate according to both content and confidence judgment (for similar analysis strategies, see Yonelinas et al., 2005; Woodruff et al., 2006). A potential issue related to collapsing this way is that disproportionate numbers of old and new items contribute to the different confidence judgments. As a result, the effects could indicate simple differences in old/new status rather than a linear relationship with confidence (or familiarity). To address this issue, we carried out an additional analysis in which only the old items contributing to each confidence level were used. To compensate for the reduced statistical power of the old-item familiarity analysis, due to using fewer items, a height threshold of *P* < 0.01 was employed with the original 42-voxel extent threshold. Figure 3 shows the results of this analysis. As can be seen, familiarity effects based on old items alone were evident in left intraparietal sulcus and in left middle frontal gyrus, similar to the findings of the original familiarity analysis.

Although the results of the above analyses show that the recollection and familiarity effects in lateral parietal cortex can be dissociated, they do not address the issue of whether the effects might overlap. As noted in the Introduction, overlapping effects would indicate that activity increases as judgments become more confident and again as a consequence of recollection, consistent with a strength-based account of recognition memory (Squire et al., 2007; Wixted, 2007). To test for such overlap, an additional analysis was conducted in which the outcome of the recollection contrast (R_scene_/R_sentence_ > 4/3/2/1) was inclusively masked with the results of the familiarity contrast (linear weights decreasing across 4, 3, 2, and 1). For consistency, the same thresholds and minimum cluster extents used earlier for these contrasts were implemented here. The analysis revealed no overlapping voxels (see Yonelinas et al., 2005, for similar findings).

For completeness, we also identified regions exhibiting the “reversed” recollection (R_scene_/R_sentence_ < 4/3/2/1) and familiarity (linear contrast weights of −3/−1/1/3 for the 4/3/2/1 judgments, respectively) effects, which are reported less often in episodic retrieval studies (for exceptions, see Yonelinas et al., 2005; Daselaar et al., 2006). The results of these contrasts are detailed in Table 3. In the context of our hypotheses for the current study, these reversed contrasts were not as relevant as were the standard recollection and familiarity contrasts; thus, we did not carry out any further exclusive masking of the results. Notably, a reversed recollection effect was identified in left intraparietal sulcus, among other regions, consistent with the non-monotonic results that are described in more detail in the next section.

**Table 3:** **Regions exhibiting “reversed” recollection and familiarity effects**.

**Region**	**BA**	**Number of voxels**	**Peak coordinates**	**Peak Z score**
**REVERSED RECOLLECTION EFFECTS**				
L superior frontal sulcus	6/8	261	−26, −5, 47	4.95
R intraparietal sulcus	7/40	3269	51, −30, 46	4.76
L intraparietal sulcus	7/40	1031	−46, −28, 42	4.67
L middle frontal gyrus	9/46	256	−58, 2, 39	4.66
R middle frontal gyrus	9/46	359	54, 5, 35	4.64
R superior frontal sulcus	6/8	252	23, 2, 53	4.25
L cingulate/superior frontal gyrus	32/8	101	−4, 0, 49	3.69
R inferior frontal gyrus	44/45	56	46, 19, 0	3.67
R intraparietal sulcus (posterior)	39/7	59	30, −63, 32	3.56
R inferior parietal lobule	39	53	35, −75, 35	3.36
**REVERSED FAMILIARITY EFFECTS**				
L lateral sulcus (posterior)	40/41	52	−60, −26, 14	4.23
L postcentral	1/2/3	109	−35, −19, 60	4.19
R lateral sulcus (anterior)	6/22	44	53, −4, 5	3.70

Note that the analyses identifying these effects did not involve exclusive masking (as opposed to those reported in Table 2; see text for further details). Peak coordinates (x, y, z) are in MNI space and rounded to the nearest mm. BA, Brodmann area (approximate); L, left; R, right.

#### Non-monotonic effects

**Figure 4:**
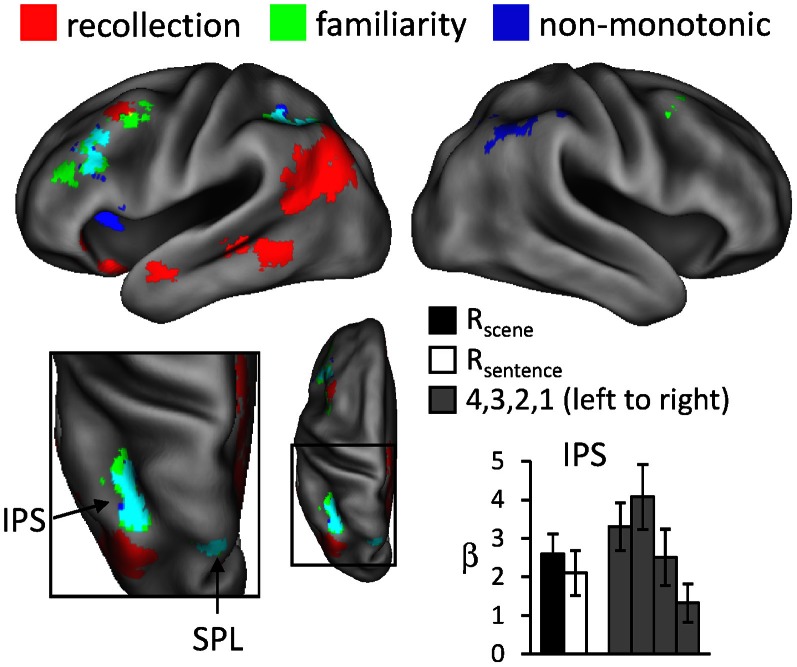
**Regions exhibiting non-monotonic effects (blue), as identified by greater activity for 4 compared to R and 1 judgments (see text for details), across the test-phase judgments.** The neural correlates of recollection (red) and familiarity (green) from previous figures are also displayed. Arrows point to overlap (in cyan) of non-monotonic and familiarity effects in intraparietal sulcus (IPS) and superior parietal lobule (SPL). The histogram indicates mean parameter estimates (β) from the peak voxel of the monotonic effect in IPS. R (remember); 4, 3, 2, 1 (confidence judgments).

For the reason outlined in the Introduction, we hypothesized that regions sensitive to familiarity would exhibit higher activity for items recognized with high-confidence than for recollected items. We tested this prediction with a direct contrast of non-monotonic effects across the 1, 4, and R judgments (contrast weights of -2/4/-1/-1 for the 1/4/R_scene_/R_sentence_ judgments, respectively). That is, the contrast identified regions where activity increased with familiarity (from 1 to 4 judgments) but then decreased in the presence of recollection. Importantly, because aspects of familiarity and the recollection-related reduction were combined into a single contrast (rather than via inclusive masking), this analysis was statistically independent of the familiarity analysis conducted earlier, and thus the same thresholding procedure (*P* < 0.005 for 42 contiguous voxels) was employed. The results of this analysis, which are shown in Figure 4 and listed in Table 4, revealed effects in several regions. Of particular interest is the correspondence between these effects and the regions that were associated with familiarity in the analyses described above. As shown in Figure 4, non-monotonic effects in left intraparietal sulcus overlapped extensively with the familiarity-sensitive voxels in that region. Also shown in the figure are the mean parameter estimates extracted from the voxel in intraparietal sulcus that exhibited the largest non-monotonic effect. Although the activity for 3 (unconfident old) judgments at this peak voxel was numerically higher than that for 4 judgments, this difference was not significant [*t*_(15)_ = 1.23, *P* = 0.238]. By comparison, the difference in activity for 4 vs. R judgments at the peak voxel in intraparietal sulcus that demonstrated the familiarity effect (see Figure 1) gave rise to a significant effect, *t*_(15)_ = 1.79, *P* < 0.05 (one-tailed).^3^ Overlapping effects were also evident in left superior parietal lobule and left lateral PFC (see Table 4 for details).

**Table 4:** **Regions exhibiting non-monotonic effects across test-phase judgments**.

**Region**	**BA**	**Number of voxels**	**Peak coordinates**	**Peak Z score**
**NON-MONOTONIC EFFECTS**				
R intraparietal sulcus	7/40	72	37, −51, 39	4.65
L middle frontal gyrus	9/46	81	−46, 25, 28	4.35
L inferior frontal gyrus	44/45	93	−40, 19, 9	3.99
L superior parietal lobule	7	56	−12, −72, 51	3.97
R intraparietal sulcus	7/40	51	53, −35, 51	3.86
R inferior parietal lobule	39/40	131	51, −58, 46	3.81
L intraparietal sulcus	7/40	104	−37, −53, 44	3.69
L middle frontal gyrus	9/46	70	−44, 26, 39	3.64
R precuneus	7	61	4, −60, 53	3.36
**OVERLAP OF NON-MONOTONIC EFFECTS WITH FAMILIARITY EFFECTS**				
L middle frontal gyrus	9/46	50	−46, 25, 28	4.35
L superior parietal lobule	7	46	−12, −72, 51	3.97
L intraparietal sulcus	7/40	75	−37, −53, 44	3.69
L middle frontal gyrus	9/46	55	−44, 26, 39	3.64

Peak coordinates (x, y, z) are in MNI space and rounded to the nearest mm. BA, Brodmann area (approximate); L, left; R, right.

#### Content-sensitive effects

**Figure 5:**
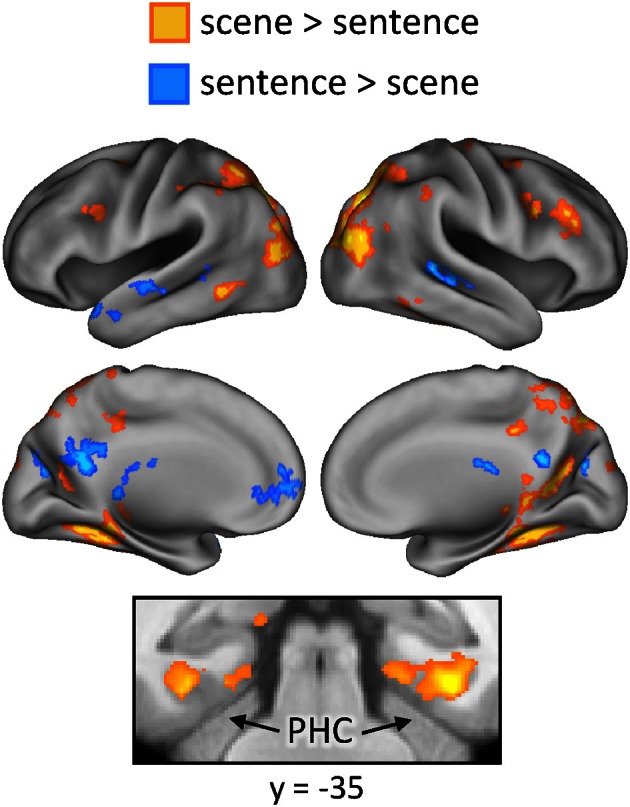
**Regions exhibiting content-sensitive differences during the study phase.** Warm colors indicate enhanced activity for the scene condition; cool colors indicate enhanced activity for the sentence condition. PHC (parahippocampal cortex).

Finally, we investigated whether the neural correlates of recollection were sensitive to the manipulation of studied content. First, regions exhibiting content-sensitive differences at the time of study were identified by directionally contrasting activity across the two content conditions (scene > sentence, and sentence > scene). The results of these contrasts are displayed in Figure 5. Greater activity for the scene study condition was evident bilaterally in widespread areas of lateral and superior parietal cortex, retrosplenial cortex, precuneus, and posterior parahippocampal cortex. Sentence-related enhancement, on the other hand, was evident in ventromedial PFC, lateral temporal cortex, and a medial posterior region that extended from posterior cingulate to cuneus.

**Figure 6:**
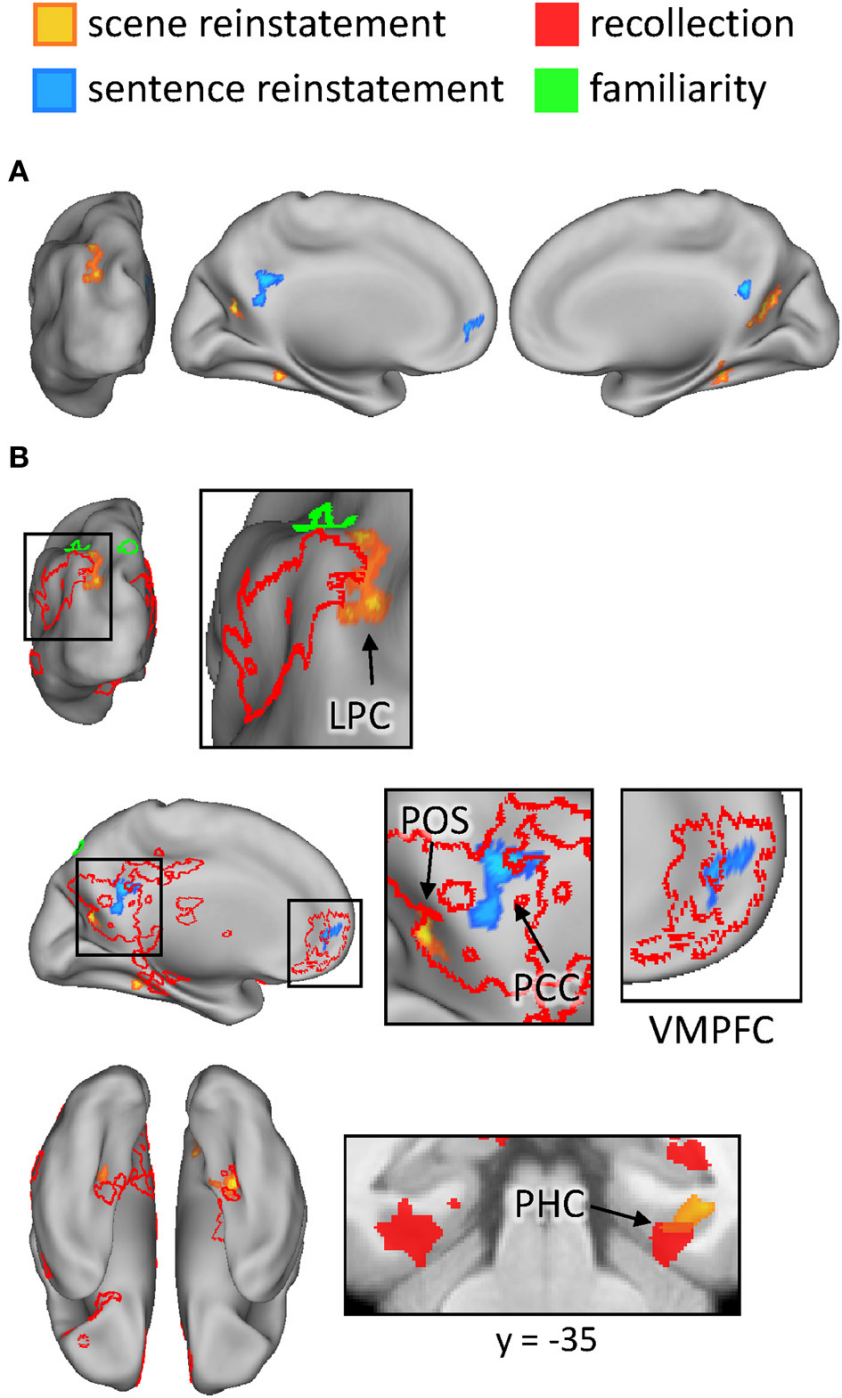
**(A)** Regions exhibiting content-sensitive reinstatement effects. Warm colors indicate reinstatement-related activity for the scene condition (scene > sentence at study, inclusively masked with R_scene_ > R_sentence_ at test); cool colors indicate reinstatement-related activity for the sentence condition (sentence > scene at study, inclusively masked with R_sentence_ > R_scene_ at test). **(B)** The content-sensitive reinstatement effects are displayed alongside the neural correlates of recollection (red outline and solid) and familiarity (green outline and solid). Arrows point to notable overlapping and adjacent effects that are described further in the text. LPC (lateral parietal cortex), POS (parieto-occipital sulcus), PCC (posterior cingulate cortex), VMPFC (ventromedial prefrontal cortex), PHC (parahippocampal cortex).

The outcomes of the aforementioned study-phase contrasts (e.g., scene > sentence) were then inclusively masked with the analogous contrasts for recollected test items ([R_scene_ > R_sentence_], in this example). The test-phase contrasts were thresholded at *P* < 0.05 and combined with the statistically-independent study-phase contrasts (at *P* < 0.005) to give a conjoint threshold of *P* < 0.0023 (Fisher, 1950; Lazar et al., 2002). This conjoint threshold was used along with the minimum cluster extent of 42 voxels that was applied to the previous analyses. The results of this masking procedure, for each direction of the content comparison, are shown in Figure 6A and listed in Table 5. Although several regions were identified by this procedure, we were especially interested in the extent to which these reinstatement effects overlapped with the generic recollection and familiarity effects identified earlier. Figure 6B displays the regions exhibiting overlapping effects (also see Table 5). Scene reinstatement effects in right parahippocampal cortex and left medial parieto-occipital cortex overlapped with recollection effects in these regions. Similarly, the sentence reinstatement effect in posterior cingulate overlapped with recollection-sensitive voxels. There was, however, minimal overlap between the reinstatement effects and the recollection and familiarity effects in lateral parietal cortex, with the reinstatement effects being localized more posteriorly along the vertical bank of the intraparietal sulcus.

**Table 5:** **Regions exhibiting content-sensitive reinstatement effects**.

**Region**	**BA**	**Number of voxels**	**Peak coordinates**	**Peak Z score**
**SCENE REINSTATEMENT**				
R medial parieto-occipital sulcus	31	186	21, −54, 14	5.16
R posterior parahippocampal cortex	36	168	28, −39, −12	4.87
L posterior parahippocampal cortex	36	43	−32, −40, −16	4.74
L medial parieto-occipital sulcus	31	65	−19, −56, 16	4.37
L posterior intraparietal sulcus	7/19	176	−30, −74, 28	4.23
**SENTENCE REINSTATEMENT**				
L posterior cingulate	31	195	−2, −56, 33	4.65
L medial prefrontal cortex^a^	32	41	−7, 61, 2	3.90
**OVERLAP OF SCENE REINSTATEMENT WITH CORE RECOLLECTION EFFECTS**				
R posterior parahippocampal cortex	36	32	32, −35, −18	4.80
L medial parieto-occipital sulcus	31	19	−18, −56, 18	4.06
**OVERLAP OF SENTENCE REINSTATEMENT WITH CORE RECOLLECTION EFFECTS**				
L posterior cingulate	31	63	−7, −54, 19	4.62
**OVERLAP OF SCENE REINSTATEMENT WITH SCENE-SPECIFIC RECOLLECTION EFFECTS**				
R posterior parahippocampal cortex	36	59	32, −35, −18	4.80
L medial parieto-occipital sulcus	31	50	−18, −56, 16	4.37
**OVERLAP OF SENTENCE REINSTATEMENT WITH SENTENCE-SPECIFIC RECOLLECTION EFFECTS**				
L posterior cingulate	31	123	−2, −56, 33	4.65
L medial prefrontal cortex^b^	32	52	−9, 51, −7	3.67

Peak coordinates (x, y, z) are in MNI space and rounded to the nearest mm. Peak coordinates and peak Z scores correspond to the study-phase contrast, although note that these results were further inclusively masked with the analogous test-phase contrasts. BA, Brodmann area (approximate); L, left; R, right.

a This effect was significant only when decreasing the extent threshold by one voxel.

b This effect was comprised of three clusters totaling 52 voxels (see text).

It is worth noting a difference between the reinstatement analyses conducted here and those conducted in our previous study (Johnson and Rugg, 2007). In that study, although we were interested in reinstatement during recollection, we were not interested in whether such reinstatement effects were evident in the ‘core recollection network’. Thus, in that study we only masked the content-sensitive study-test overlap with recollection-related activity that was specific to the content condition of interest (as opposed to recollection-related activity that generalized across both conditions, as was described above for the current analyses). To facilitate comparison between the present and previous studies, we conducted an additional analysis of the current data. This analysis involved using the outcomes of the foregoing study-test masking procedures and further applying an inclusive mask corresponding to the content-specific recollection contrast for the condition of interest ([R_scene_ > 4/3/2/1] for scene reinstatement, and [R_sentence_ > 4/3/2/1] for sentence reinstatement; *P* < 0.005). The results of this analysis are listed in Table 5. As is apparent in the table, the same regions reported above as exhibiting overlap with the core recollection effects were again evident. Note that, to reveal the sentence reinstatement effect in ventro-medial PFC, which was evident in our earlier analysis and in our previous study, it was necessary to reduce the minimum extent threshold; doing so resulted in three clusters of significant voxels in this region, totaling 52 voxels.

## Discussion

Before turning to the fMRI findings, we first address relevant aspects of the behavioral results. First, as was reported in two prior studies that employed the same encoding tasks (Johnson and Rugg, 2007; Johnson et al., 2008a), the probability of recollection (as indexed by R judgments) was greater for the sentence task than for the scene task (see Table 1). A measure of memory accuracy computed for the present data also confirmed this disparity, with higher accuracy for items from the sentence task (see section Behavioral results). These results raise the possibility that differences in recollection-related activity according to encoding task might reflect differences between the memory strengths of the two classes of test item, rather than in retrieved content. We cannot rule out this possibility in the case of regions where recollection effects took the form of greater activity for items from the sentence task. However, the majority of content-sensitive recollection effects exhibited enhanced activity for items from the scene task (see Table 5), for which this account obviously does not apply. Second, memory accuracy was considerably higher for R judgments than for 4 judgments. Whereas this difference raises the possibility that our so-called ‘recollection effects’ are instead the result of a memory strength confound, the same argument alone cannot account for the finding that other regions exhibited higher activity for items designated with 4 relative to R judgments. We return to this issue below.

Analysis of the fMRI data revealed recollection-sensitive effects in several regions, including left ventral parietal cortex, retrosplenial and posterior cingulate cortex, ventromedial PFC, bilateral hippocampus, and bilateral posterior parahippocampal cortex (for similar findings, see Henson et al., 1999; Eldridge et al., 2000; Wheeler and Buckner, 2004; Woodruff et al., 2005; Yonelinas et al., 2005; for reviews, see Spaniol et al., 2009, and Kim, 2010). As was reported previously (Yonelinas et al., 2005), this putative ‘core recollection network’ (cf. Johnson and Rugg, 2007, and Hayama et al., 2012) demonstrated no overlap with regions where activity co-varied with recognition confidence (and presumably, with familiarity), including left intraparietal sulcus, precuneus, anterior cingulate, and dorsolateral PFC. The regional dissociation between these two patterns of activity is thus consistent with models of recognition memory that propose that familiarity- and recollection-based judgments rely on neurally (and hence functionally) distinct memory signals (cf. Yonelinas, 2002; Wixted and Mickes, 2010; see below for further discussion).

The dissociation between recollection and recognition confidence effects is particularly striking within the left lateral parietal cortex. Recollection-sensitive voxels were localized to the ventral aspect of this region, in the vicinity of the angular gyrus, whereas voxels sensitive to confidence were situated more dorsally within the intraparietal sulcus. This dissociation is consistent with the findings of resting-state functional-connectivity studies (Vincent et al., 2008; Andrews-Hanna et al., 2009; Nelson et al., 2010). Resting-state activity in the angular gyrus has been shown to be coupled with activity in regions of posterior cingulate, retrosplenial cortex, hippocampus, posterior parahippocampal cortex, and ventromedial PFC—a network that is collectively referred to as the “hippocampal-cortical memory system” (Vincent et al., 2008). Activity in the intraparietal sulcus, by comparison, appears to be coupled with that in lateral PFC, precuneus, and anterior cingulate cortex as part of the “fronto-parietal control system” (Vincent et al., 2008). Yet, despite this consistency, the functional significance of this parietal dissociation is currently the subject of debate (see Wagner et al., 2005; Cabeza et al., 2008; Vilberg and Rugg, 2008; Hutchinson et al., 2009). According to one proposal (Cabeza et al., 2008; Ciaramelli et al., 2008), the dissociation reflects the engagement of two different classes of attentional processes (Corbetta and Shulman, 2002; Corbetta et al., 2008). Whereas retrieval effects in ventral parietal cortex reflect the ‘bottom-up’ re-orienting of attention triggered by the occurrence of recollection, effects in the vicinity of the intraparietal sulcus reflect ‘top-down’ attentional control processes engaged in proportion to the effort required to process a retrieval cue. According to an alternative proposal (Vilberg and Rugg, 2007, 2008), ventral parietal (more specifically, angular gyrus) activity reflects the engagement of processes supporting the representation or maintenance of recollected information, possibly akin to an episodic buffer (Baddeley, 2000). By this same account, retrieval effects in the intraparietal sulcus reflect the accumulation of evidence that a test item is old, correlating either with familiarity directly or with a more generic memory strength signal derived from a combination of familiarity and recollection.

In relation to the aforementioned accounts, the present findings shed little additional light on the functional interpretation of recollection-related activity in the angular gyrus (but see Yu et al., 2012a; Vilberg and Rugg, 2012). In two ways, however, the findings help to elucidate the significance of retrieval-related activity in the intraparietal sulcus. First, the finding that activity in intraparietal sulcus co-varied with confidence that the eliciting item was studied (see Yonelinas et al., 2005, for equivalent findings) is difficult to reconcile with an account framed in terms of top-down attention. As was noted by Cabeza et al. (2008), the attentional account predicts that activity would be maximal for items (whether judged old or new) that attracted low rather than high confidence judgments. Consequently, this account predicts a non-monotonic (inverted U-shaped) relationship in the intraparietal sulcus with recognition confidence, mirroring the pattern of RTs observed across these judgments (see Table 1). Together with the findings of Yonelinas et al. (2005), the present finding of a linear pattern of familiarity-related activity in intraparietal sulcus strongly suggests that this region is sensitive to a continuously-varying memory signal.

The second aspect of the present findings that helps elucidate the role of the intraparietal sulcus in recognition memory is the finding that recollected items elicited lower activity than did items recognized with high confidence. To our knowledge, this finding is novel in the domain of fMRI. As was discussed in the Introduction, given the assumptions underlying a widely accepted dual-process model of recognition memory (Yonelinas, 2001), the mean familiarity of recollected test items should be lower than the mean familiarity of items judged old on the basis of familiarity alone. By this argument, regions should exist where activity elicited by recollected items is lower than the activity associated with highly familiar items. We were able to identify several such regions in the present study, including an area in the left intraparietal sulcus (see Figure 4 and Table 4). Together with recent MEG findings demonstrating a neural correlate of familiarity that was larger for “know” than “remember” judgments (Evans and Wilding, 2012), the present findings strongly suggest that this (and other) regions exhibiting these non-monotonic effects may play a selective role in familiarity-driven recognition. More generally, the existence of regions where activity is maximal for highly familiar items and significantly lower for recollected items is inconsistent with models of recognition memory in which recollection and familiarity are held to differ solely in terms of the strength of a common memory signal (e.g., Donaldson, 1996; Dunn, 2004; Rotello et al., 2005).

In as much as could be determined with the restricted field of view employed in the present study (a consequence of acquiring high-resolution fMRI data), the findings for reinstatement effects replicate the results of our prior study that employed the same two study tasks (Johnson and Rugg, 2007). In both studies, reinstatement effects were evident for the sentence task in ventromedial PFC. Likewise, scene-related reinstatement was evident in right posterior parahippocampal cortex, in a region almost homologous with a left-lateralized effect reported in the previous study (peak coordinates of [32, −37, −18] versus [−27, −42, −21]). In addition to these effects, the present study also identified a double-dissociation between the two classes of reinstatement effects in medial posterior cortex. Whereas sentence-related reinstatement was evident in the posterior cingulate, scene-related reinstatement was localized more posteriorly in the medial parieto-occipital sulcus. The observation of this dissociation among nearby regions, in the absence of a similar effect in our previous study, may emphasize the benefits of acquiring fMRI data at a high spatial resolution and using optimized across-subject alignment techniques (for analogous findings in the vicinity of this area, see Johnson et al., 2009, which employed pattern-classification analyses).

As noted in the Introduction, a central aim of the present study was to investigate the extent to which the regions consistently reported to be recollection-sensitive in prior studies (see Spaniol et al., 2009, and Kim, 2010) are sensitive to the nature of the retrieved content. Whereas some recollection-related clusters (in posterior midline and parahippocampal cortex) did demonstrate content-sensitivity (see Figure 6B), the great majority of recollection-sensitive voxels were invariant with respect to content. Among these content-insensitive regions were the left intraparietal sulcus and angular gyrus (as well as the hippocampus, as discussed below). As is evident from Figure 6B, scene-related reinstatement effects abutted but shared only a minimal number of voxels with the recollection and familiarity effects in lateral parietal cortex.^4^ The present findings converge with prior evidence (Vilberg and Rugg, 2007, 2009b; Guerin and Miller, 2009; Duarte et al., 2011) to suggest that retrieval-related activity in posterior parietal cortex reflects the engagement of content-independent processes (although see Klostermann et al., 2009). Of course, it is not possible to rule out the possibility that a different (or perhaps, stronger) encoding manipulation would reveal content-sensitive effects in these regions. Additionally, as noted previously, this conclusion rests on the (arguably unwarranted) assumption that content-sensitivity in this region would take the form of differences in the mean level of activation, as afforded by standard fMRI analysis of smoothed data. It remains to be determined whether more sensitive techniques, such as pattern-classification analyses (Norman et al., 2006), could detect more subtle forms of content-sensitivity in these core recollection regions. Nonetheless, the present findings are consistent with the proposal that recollection-related activity in the vicinity of the angular gyrus reflects processes that represent retrieved content in an amodal or multi-modal form (Vilberg and Rugg, 2008; Shimamura, 2011).

Although bilateral regions of the hippocampus demonstrated recollection-related activity, consistent with its role in the core recollection network (cf. Johnson and Rugg, 2007; Hayama et al., 2012), content-sensitive effects were absent throughout the hippocampus. The failure to find content effects in this region is somewhat surprising given that, even at the rather broad level of laterality, neuropsychological studies tend to show a left-right asymmetry for verbal versus spatial tasks (e.g., Smith and Milner, 1981; Frisk and Milner, 1990; but see Spiers et al., 2001). This verbal-spatial distinction arguably maps well onto the sentence versus scene conditions employed here. At a finer level, the evidence for content-sensitivity within the hippocampus is somewhat mixed. On one hand, some recent studies have shown the hippocampus to be insensitive to simple manipulations of stimulus material (e.g., Diana et al., 2008; Liang et al., 2013; also see Ritchey et al., in press). Such evidence is consistent with the idea that the hippocampus binds together different types of information—such as item and contextual information processed in perirhinal and parahippocampal regions, respectively—into ‘domain-general’ representations that are employed for episodic memory (Diana et al., 2007; Eichenbaum et al., 2007; also see Davachi, 2006). On the other hand, studies making use of stimuli with much more elaborate spatial and temporal characteristics, such as film clips and virtual-reality environments, have demonstrated that the hippocampus can indeed distinguish between individual episodic memories that differ richly in content (e.g., Hassabis et al., 2009; Chadwick et al., 2010). Notably, each of these studies employed pattern-classification analyses, thereby placing emphasis on whether the hippocampus as a whole can discriminate between different experimental conditions. It remains to be determined whether content-sensitive recollection effects within the hippocampus are dissociable in terms of their loci, as might be expected on the basis of evidence indicating that anterior and posterior hippocampal regions demonstrate different patterns of functional connectivity (e.g., Libby et al., 2012; also see Ranganath and Ritchey, 2012).

Before concluding, we briefly discuss an alternative account of the proposal—arguably consistent with the present findings—that familiarity- and recollection-related recognition memory can be dissociated by virtue of their different fMRI response profiles. This account (Squire et al., 2007; also see Song et al., 2011) argues that the dissociation is due to regional differences in the transfer function between neural activity and the ensuing BOLD signal. By this account, familiarity- and recollection-based recognition differ not because they are supported by qualitatively-distinct memory signals, but because they reflect differences in the strength of a common signal. Regions where the BOLD signal is ‘familiarity-sensitive’ are those where the BOLD response asymptotes at relatively low levels of neuronal firing (and relatively low levels of memory strength), thus leading to the response being insensitive to the strengths of highly-familiar versus recollected test items. By contrast, regions where the BOLD response is insensitive to low levels of neural activity, and is enhanced only when activity (and memory strength) is high, lead to the appearance of a ‘recollection-sensitive’ effect. Data inconsistent with this proposal have been reported previously (e.g., Diana et al., 2009; Rugg et al., 2012; Yu et al., 2012b). Whereas the present findings do not add to the evidence weighing against this account as it applies to recollection-sensitive effects, they add substantially to the evidence opposing its validity with respect to regions putatively sensitive to familiarity. As already discussed, we found significant overlap between familiarity-sensitive regions (as identified by a monotonic relationship between the BOLD signal and recognition confidence) and regions where activity was lower for recollected than for highly familiar test items (also see Evans and Wilding, 2012). This finding is incompatible with an account of familiarity-sensitive effects in terms of a ‘saturating’ BOLD signal as well as the proposal that recollection and familiarity differ solely in terms of the strength of a common memory signal.

To conclude, the present study provides a high-resolution characterization of activity associated with familiarity- and recollection-driven recognition memory. The findings strongly support prior proposals that these two forms of recognition memory are neurally and, hence, functionally distinct. Additionally, they add to the evidence for the existence of a content-insensitive core recollection network that includes the angular gyrus, retrosplenial and posterior cingulate cortex, ventromedial PFC, hippocampus, and posterior parahippocampal cortex (cf. Hayama et al., 2012). Finally, the findings add to the evidence that recollection involves the engagement of this core network in conjunction with cortical regions in which the contents of recollection are represented by the reinstatement of encoding-related activity (Rugg et al., 2008; Rissman and Wagner, 2012). An important goal for the future is to understand how these two facets of recollection-related neural activity interact so as to permit retrieved episodic information to inform current behavioral goals.

### Conflict of interest statement

The authors declare that the research was conducted in the absence of any commercial or financial relationships that could be construed as a potential conflict of interest.

## Footnotes

^1^Here, we use the term ‘contents’ to refer to the information inherently associated with an item (e.g., its color or size), as well as the context in which the item is encoded (e.g., spatiotemporal information or the encoding task performed)—in other words, any qualitative information that might be subsequently recollected.

^2^In preliminary analyses, we conducted additional contrasts that compared R judgments with 4 judgments alone and with the combination of 4 and 3 judgments. Because each of these analyses gave rise to a similar pattern of results, and since we ultimately exclusively masked out any differences that were evident across confidence judgments, we chose to report only the R > 4/3/2/1 results here.

^3^Note that the latter comparison comes from the familiarity-sensitive peak and is thus independent of the non-monotonic contrast employed to identify this region.

^4^In a previous study (Johnson et al., 2009), which employed pattern-classification analyses along with a different set of encoding conditions than used here, we also observed a reinstatement effect in left lateral parietal cortex. Relative to the loci of the recollection and familiarity effects observed here, the reinstatement effect in that previous study appears considerably more ventral (peak coordinates: −57, −45, −9), consistent with the notion that these different types of effects are anatomically dissociable.
